# Vitamin D Attenuates Ischemia/Reperfusion-Induced Cardiac Injury by Reducing Mitochondrial Fission and Mitophagy

**DOI:** 10.3389/fphar.2020.604700

**Published:** 2020-12-10

**Authors:** Tzu-Lin Lee, Ming-Hsueh Lee, Yu-Chen Chen, Yi-Chieh Lee, Tsai-Chun Lai, Hugo You-Hsien Lin, Lee-Fen Hsu, Hsin-Ching Sung, Chiang-Wen Lee, Yuh-Lien Chen

**Affiliations:** ^1^ Department of Anatomy and Cell Biology, College of Medicine, National Taiwan University, Taipei, Taiwan; ^2^ Division of Neurosurgery, Department of Surgery, Chang Gung Memorial Hospital, Chiayi, Taiwan; ^3^ Department of Respiratory Care, Chang Gung University of Science and Technology, Chiayi, Taiwan; ^4^ Division of Nephrology, Department of Internal Medicine, Kaohsiung Medical University Hospital, Kaohsiung, Taiwan; ^5^ Department of Anatomy, College of Medicine, Chang Gung University, Taoyuan, Taiwan; ^6^ Aesthetic Medical Center, Department of Dermatology, Chang Gung Memorial Hospital, Taoyuan, Taiwan; ^7^ Department of Nursing, Division of Basic Medical Sciences, and Chronic Diseases and Health Promotion Research Center, Chang Gung University of Science and Technology, Chiayi, Taiwan; ^8^ Research Center for Industry of Human Ecology and Research Center for Chinese Herbal Medicine, Chang Gung University of Science and Technology, Taoyuan, Taiwan; ^9^ Department of Orthopaedic Surgery, Chang Gung Memorial Hospital, Chiayi, Taiwan

**Keywords:** cardiac ischemia/reperfusion, mitochondrial fission, vitamin D3, apoptosis, mitophagy

## Abstract

Myocardial infarction is the leading cause of morbidity and mortality worldwide. Although myocardial reperfusion after ischemia (I/R) is an effective method to save ischemic myocardium, it can cause adverse reactions, including increased oxidative stress and cardiomyocyte apoptosis. Mitochondrial fission and mitophagy are essential factors for mitochondrial quality control, but whether they play key roles in cardiac I/R injury remains unknown. New pharmacological or molecular interventions to alleviate reperfusion injury are currently considered desirable therapies. Vitamin D_3_ (Vit D_3_) regulates cardiovascular function, but its physiological role in I/R-exposed hearts, especially its effects on mitochondrial homeostasis, remains unclear. An *in vitro* hypoxia/reoxygenation (H/R) model was established in H9c2 cells to simulate myocardial I/R injury. H/R treatment significantly reduced H9c2 cell viability, increased apoptosis, and activated caspase 3. In addition, H/R treatment increased mitochondrial fission, as manifested by increased expression of phosphorylated dynein-related protein 1 (p-Drp1) and mitochondrial fission factor (Mff) as well as increased mitochondrial translocation of Drp1. Treatment with the mitochondrial reactive oxygen species scavenger MitoTEMPO increased cell viability and decreased mitochondrial fission. H/R conditions elicited excessive mitophagy, as indicated by increased expression of BCL2-interacting protein 3 (BNIP3) and light chain (LC3BII/I) and increased formation of autolysosomes. In contrast, Vit D_3_ reversed these effects. In a mouse model of I/R, apoptosis, mitochondrial fission, and mitophagy were induced. Vit D_3_ treatment mitigated apoptosis, mitochondrial fission, mitophagy, and myocardial ultrastructural abnormalities. The results indicate that Vit D_3_ exerts cardioprotective effects against I/R cardiac injury by protecting mitochondrial structural and functional integrity and reducing mitophagy.

## Introduction

The World Health Organization (WHO) reports that acute myocardial infarction is the leading cause of morbidity and mortality in many regions of the world (Roth et al., 2017). Timely reperfusion is the most effective approach to save ischemic myocardium. However, reperfusion can induce ischemia-reperfusion (I/R) injury. I/R injury is a multifactorial pathophysiological process that causes cell damage during hypoxia, and the damage becomes more severe when oxygen is re-delivered into the tissue (Rossello et al., 2018). In addition, I/R causes a series of adverse events, such as excessive reactive oxygen species (ROS) production, calcium overload, inflammatory responses, increased apoptosis, and mitochondrial dysfunction, all of which lead to myocardial cell death and accelerate myocardial damage (Yellon and Hausenloy, 2007). Despite the clinical importance of I/R injury, bedside treatments that inhibit I/R are still limited, mainly due to the complex mechanisms that contribute to I/R. New pharmacological or molecular interventions that alleviate reperfusion injury are highly desirable for current reperfusion therapy (Baehr et al., 2019; Yu et al., 2019).

Oxidative stress is a result of increased ROS levels. ROS are produced by mitochondria due to aerobic metabolism during I/R (Chouchani et al., 2014). The cytotoxicity of ROS is associated with the rapid modification of cellular components, including the reduced ability to produce ATP (Navarro and Boveris, 2007). Excessive production of ROS causes toxicity by disrupting the electron transport chain and interfering with mitochondrial permeability transition pores, leading to apoptosis or necrosis (Hausenloy and Yellon, 2013). Although studies have shown that ROS play a key role in heart tissue damage, there is currently no effective treatment. Further research may provide new insights into the clinical treatment of I/R.

Maintaining mitochondrial function and integrity plays a crucial role in normal cell physiology, especially in cardiomyocytes with high energy requirements (Hall et al., 2014). In addition to producing ATP, mitochondria are also the major source of ROS, which can trigger oxidative stress and affect cell fate (Vasquez-Trincado et al., 2016). Therefore, strict quality control mechanisms are required to maintain healthy mitochondria. These quality control mechanisms primarily include mitochondrial dynamics, fission and fusion, and mitophagy. Mitochondrial fission is usually the separation of damaged components from the original mitochondrion, resulting in one functional mitochondrion and another impaired mitochondrion (Westermann, 2010). Mitochondrial fission involves a cytoplasmic protein, dynein-related protein 1 (p-Drp1), that binds to mitochondrial fission factor (Mff) and localizes to the mitochondrial outer membrane, forming a complex that allows mitochondrial fission. However, oxidative stress causes excessive mitochondrial fission, leading to mitochondrial structural changes and dysfunction, as well as cellular damage. In addition to mitochondrial fission, mitophagy, which is a selective form of autophagy, is another specific mechanism by which dysfunctional or impaired mitochondria are degraded and recycled; mitophagy maintains healthy mitochondrial populations and mitochondrial quality (Ni et al., 2015). Mitochondria that are isolated by mitochondrial fission are cleared by mitophagy, and mitophagy plays a crucial role in maintaining mitochondrial homeostasis (Mao and Klionsky, 2013; Zhou et al., 2020). Mitophagy can maintain energy metabolism in the body to a certain extent and reduce damage caused by external stimuli, thereby protecting the human body; under normal conditions, cellular mitophagy is low (Ravikumar et al., 2010). However, excessive mitophagy can lead to cellular injury (Yang et al., 2019). This observation suggests that mitophagy is regulated by receptor-mediated mitophagy (Schiattarella and Hill, 2016; Bravo-San Pedro et al., 2017). BCL2/adenovirus E1B 19-kDa protein-interacting protein 3 (BNIP3) is localized to the mitochondrial outer membrane and is a receptor-related factor required for mitochondrial elimination. BNIP3 interacts with the LC3 protein family through its cytosol-directed LIR motif, thus mediating mitophagy. However, the role of BNIP3 and LC3B and the role of mitophagy during I/R injury are unclear.

Patients suffering from cardiovascular diseases are frequently deficient in vitamin D (Dibaba, 2019). Previous reports have shown that vitamin D_3_ metabolites [including 25-hydroxyvitamin D_3_ (25(OH)D_3_) and 1α,25-dihydroxyvitamin D_3_ (1α,25(OH)_2_D_3_)] affect the uptake of calcium and phosphorus, the growth of cells, and the expression of many genes in skeletal muscle cells and cultured myoblast cell lines (Alfawaz et al., 2014; Tao et al., 2015). Low serum levels of 25(OH)D_3_ in patients can cause a significantly higher risk of death from heart failure (Liu et al., 2016). Vitamin D is closely associated with cardiac hypertrophy and fibrosis and with atherosclerosis development (Artaza et al., 2009; Gardner et al., 2013). Supplementation with a high dose of vitamin D (25(OH)D_3_, 4000 IU/day) for 12 months can improve the left ventricular ejection fraction and reverse left ventricular remodeling in patients with heart failure and vitamin D deficiency (25(OH)D_3_ < 20 ng/ml) (Witte et al., 2016). Although there is evidence that vitamin D is associated with heart disease, little information is available regarding 25-hydroxyvitamin D_3_ (Vit D) and I/R. In addition, the role of mitophagy during Vit D treatment in preventing I/R injury remains unclear. Using I/R-exposed mice and H/R-treated cells, we will 1) examine the levels of apoptosis and ROS, 2) elucidate the role of Drp-1 and Mff in mitochondrial dynamics, and 3) identify the role of BNIP3 and LC3B in mitophagy during the progression of mitochondrial dysfunction in I/R-exposed mice treated with Vit D.

## Materials and Methods

### Cell Culture and Hypoxia/Reoxygenation Procedure

H9c2 cells, which are rat embryonic ventricular cardiomyocytes, were obtained from American Type Culture Collection (ATCC, VA, USA). The H9c2 cells were cultured in Dulbecco’s modified Eagle’s medium (Gibco, MA, USA) containing 10% fetal bovine serum [Biological Industries (BI), CT, USA] and supplemented with 1% penicillin/streptomycin/amphotericin B and 2 mM L-glutamine (BI). The cells were maintained in a humidified incubator at 37 °C in 5% CO_2_ and 95% air.

H9c2 cells were pretreated for 4 h with or without 100 nM 25-hydroxyvitamin D3 (Vit D) (Cayman, UM, USA) or 10 nM MitoTEMPO (Santa Cruz, TX, USA). In addition, to evaluate the effect of mitochondrial fission or autophagy on mitophagy, H9c2 cells were pretreated for 4 h with or without the mitochondrial fission inhibitor, Mdivi-1 (10 µM) or the autophagy inhibitor, bafilomycin A1 (10 µM) (Cayman). Hypoxia and reoxygenation (H/R) was carried out based on a previously described method (Yao et al., 2019). In brief, the hypoxic cell culture medium lacked serum. The H9c2 cells were then incubated for 6 h at 37 °C in an anaerobic chamber under hypoxic conditions (1% O_2_). The cells were then transferred to a conventional incubator for 12 h. The corresponding control cells were incubated under normoxic conditions for the same duration.

### Cell Viability Assay

The cytotoxic effects of H/R and Vit D were assessed by 3-(4,5-dimethylthiazol-2-yl)-2,5-diphenyltetrazolium bromide (MTT) (Bionovas, ON, CA). After various treatments, MTT solution was added at a final concentration of 0.5 mg/ml and incubated for 4 h in 5% CO_2_ at 37 °C. The MTT-containing media were then removed, and the formazan crystals were dissolved by adding dimethyl sulfoxide (DMSO, 150 μL/well), followed by incubation for 15 min with mild shaking at room temperature (RT). The optical density was measured spectrophotometrically at 550 nm using a microplate reader (Biotek, VT, USA). The cell viability was expressed relative to that of the control.

### Terminal Deoxynucleotidyl Transferase dUTP Nick-End Labeling (TUNEL) Staining

A TUNEL apoptosis assay was used to detect DNA fragmentation using an *in situ* cell death detection kit (Roche, CA, USA) according to the manufacturer’s instructions. Briefly, H9c2 cells were fixed in 4% paraformaldehyde for 20 min after H/R stimulation for the indicated time points. The samples were then incubated with the TUNEL reagent in a dark, humidified chamber at 37 °C for 1 h. As a negative control, cells and tissues were treated only with the labeling solution. Nuclear counterstaining with 4′,6-diamidino-2-phenylindole (DAPI) (Southern Biotech, AL, USA) was performed, and the stained cells were examined using a fluorescence microscope (Leica, Wetzlar, Germany). The number of TUNEL-positive nuclei was counted under a high-power field in six different non-overlapping fields from each slide.

### Annexin V/Propidium Iodide Assay

Apoptotic and necrotic cells were quantified by annexin V-FITC binding and propidium iodide (PI) uptake (BioLegend, CA, USA) according to the provided protocols. Briefly, after the cells were treated as indicated, the cells were harvested, resuspended in 100 μL binding buffer containing 2.5 μL FITC-annexin V and 5 μL PI solution (100 μg/ml), and incubated for 15 min in the dark at 4 °C. The cellular fluorescence was measured using a FACSCalibur flow cytometer (BD, NJ, USA). The cells that were considered viable were negative for both dyes, while the cells that were in the early phase of apoptosis were annexin V-positive and PI-negative, the cells that were in late phase of apoptosis were annexin V/PI-positive, and the cells that were in necrosis annexin V-negative and PI-positive.

### Western Blot Analysis and Co-immunoprecipitation

Western blot was performed as previously described (Pu et al., 2017). Cardiac tissues and cells were homogenized in RIPA lysis buffer [50 mM Tris, pH 7.4, 150 mM NaCl, 1% NP-40, 0.5% sodium deoxycholate, 0.1% sodium dodecyl sulfate (SDS)]. Samples with equal amounts of protein (20 μg) were electrophoresed in an SDS-polyacrylamide gel and transferred to polyvinylidene fluoride (PVDF) membranes (Millipore, MA, USA). These membranes were probed overnight at 4 °C with the following primary antibodies: caspase 3, cytochrome c, p-Drp1, Mff, and LC3B, which were purchased from Cell Signaling (MA, USA), HIF-1α and Bax, which were purchased from GeneTex (Hsinchu city, Taiwan), Bcl-2, which was purchase from BD, and BNIP3, which was purchased from Aviva Systems Biology (CA, USA). Then, the membranes were incubated with a horseradish peroxidase-conjugated goat anti-mouse or anti-rabbit IgG secondary antibody (Jackson, PA, USA). The bound antibodies were detected using enhanced chemiluminescence (ECL) (Merck, NJ, USA). The intensity of the bands was quantified using ImageJ software (NIH, MD, USA). β-actin (Abcam, MA, USA) was used as the internal standard.

For co-immunoprecipitation, cells were collected and lyzed with lysis buffer. The supernatant fractions were collected and incubated with 1 μg of the appropriate antibody and precipitated overnight with protein A/G Sepharose beads (G-Bioscience, MO, USA) at 4°C. The beads were washed 3 times with wash buffer by centrifugation at 2,500 g at 4 °C. The precipitated proteins were collected by centrifugation at 2,500 g for 5 min. The immunoprecipitated proteins were separated by SDS-PAGE and subjected to Western blot as described above. The primary antibodies were as follows: Drp1 (Cell Signaling) and LC3B antibodies. The precipitation purity was also evaluated with Mff and BNIP3 antibodies.

### Analysis of Mitochondrial Reactive Oxygen Species and Cellular Reactive Oxygen Species Levels

The levels of mitochondrial ROS were detected using the mitochondrial superoxide indicator MitoSox Red (Invitrogen, MA, USA). H9c2 cells were treated with 1 μM MitoSox Red for 15 minat 37 °C. Fluorescent images were captured using a fluorescence microscope. TO-PRO-3 (100 nM, Thermo, MA, USA), a dead cell indicator, was added before MitoSox Red analysis by an LSRFortessa flow cytometer (BD). 2′,7′-Dichlorodihydrofluorescein diacetate (DCFH-DA) (Thermo) and dihydroethidium (DHE) (Invitrogen) were used to detect the levels of intracellular oxidative free radicals and superoxide anions, respectively. Cardiomyocytes on coverslips were treated with or without H/R and then incubated with serum-free medium containing DCFH-DA (10 μM) in the dark at 37 °C for 30 min. After incubation, the conversion of DCFH-DA to the fluorescent product DCF was detected by fluorescence microscopy, and 5 μL PI solution (100 μg/ml) was added before DCFH-DA analysis by flow cytometry. In addition, cardiomyocytes were incubated in serum-free medium containing DHE (5 μM) for 15 min at 37 °C. Superoxide anions oxidize DHE, yielding ethidium, which emits red fluorescence at 535 nm, and images were captured by fluorescence microscopy. DiOC_6_(3) (90 nM, Thermo), a lipophilic dye that is selective for the mitochondria of live cells, was added before DHE analysis by flow cytometry.

### Determination of Mitochondrial Membrane Potential (ΔψM)

5,5′,6,6′-Tetrachloro-1,1′,3,3′-tetraethylbenzimidazolyl-carbocyanine iodide (JC-1, BD) was used to analyze changes in mitochondrial transmembrane potential. After the various treatments, the cells were incubated with JC-1 (2 μM) at 37 °C for 30 min in the dark. JC-1 monomers emit green fluorescence and indicate the dissipation of the ΔψM, whereas JC-1 aggregates emit red and indicate an intact ΔψM. Cell fluorescence was monitored using an LSM 510 confocal microscope (Zeiss, Oberkochen, Germany) and a FACSCalibur flow cytometer.

### Adenosine Triphosphate Determination

The levels of cellular ATP were evaluated using an ATP assay kit (Molecular Probe, OR, USA). The cells were lyzed and then centrifuged (12,000 g) for 5 min at 4 °C. Subsequently, the supernatants were collected, and the ATP levels were analyzed. The ATP working reagent was added to the 96-well plate for 5 min, and 20 μL of each sample was also added to each well. The ATP levels were measured using a microplate luminometer (Berthold, Bad Wildbad, Germany) and calculated according to standard ATP curves.

### Acridine Orange Staining

Cells were briefly washed with PBS and incubated with 1 μg/ml acridine orange hydrochloride solution (Invitrogen) in PBS for 20 min at RT. Then, the cells were analyzed using confocal microscopy and flow cytometry. The acidic autophagic vacuoles emitted bright red fluorescence, while green fluorescence was observed in the cytoplasm and nucleus.

### Double Immunofluorescence Staining

After the indicated treatments, H9c2 cells on sterilized coverslips were washed with PBS, fixed with 4% paraformaldehyde for 15 min at RT, and then permeabilized with 0.1% Triton X-100 for 10 min at RT. After washing with PBS, the cells were blocked with 3% bovine serum albumin (BSA) (Novagen, Darmstadt, Germany) in PBS for 1 h at RT. The cells were incubated with primary anti-Drp1 (Cell Signaling) or anti-LC3B (Cell Signaling) (1:250 dilution in PBS containing 1% BSA) antibodies overnight at 4 °C. After washing three times with PBS, the cells were incubated with anti-rabbit Alexa Fluor® 488 (Invitrogen) (1:250 dilution in PBS containing 1% BSA) for 1 h at RT. After washing three times with PBS, the cells were incubated with COX IV (Thermo) (1:250 dilution in PBS containing 1% BSA) overnight at 4°C. After washing three times with PBS, the cells were incubated with anti-mouse Alexa Fluor® 647 (Invitrogen) antibodies for 1 h at RT. The cells were then counterstained with 1 μg/ml DAPI for 3 min. Analysis and photomicrography were performed using an LSM 510 inverted confocal microscope.

### Mitochondria Isolation Assay

A mitochondria isolation kit (Thermo) was used to prepare mitochondrial and cytoplasmic fractions from whole H9c2 cell lysates. The experimental procedures were performed according to the manufacturer’s instructions. The mitochondrial and cytoplasmic fractions were stored at −80 °C for further studies.

### Mitochondrial Morphology

Mitochondrial morphology was observed using MitoTracker staining (Invitrogen). After the different treatments, cardiomyocytes were incubated with 400 nM MitoTracker for 1 h at 37 °C in the dark. Visualization was performed using a total internal reflection fluorescence microscope (TIRF, Zeiss) along with DIC optics and epifluorescence illumination, and the length of mitochondria was measured with ImageJ software. The length of more than 50 mitochondria per cell was measured, and measurements were collected from 20 cells. Three replicates were performed for each biological sample.

### Animal Model

Adult male C57BL/6 mice (∼25 g) were obtained from National Taiwan University and housed at 25 °C ± 5 °C with 12-h light/dark cycles. All the animal experiments were performed in accordance with the National Institutes of Health guidelines for the use of laboratory animals and approved by the Taiwan University Animal Ethics Committee. The mice were randomly divided into the following groups: sham surgery, I/R, I/R + Vit D, and Vit D. One week before the surgery, Vit D was dissolved in PBS and intraperitoneally administered 3 times at 30 ng/mouse every 2 days. The sham group received PBS injections on the same schedule. The pharmacological dose was selected based on previous reports (Bodyak et al., 2007; Bae et al., 2011). In addition, bafilomycin (2.5 mg/kg) was intraperitoneally administered 1 day before I/R operation to inhibit the occurrence of autophagy. Myocardial I/R injury was performed according to the previously described method (Yang et al., 2017). Briefly, a mouse was anesthetized with 2% isoflurane, and the heart was manually exposed through a small incision without the need for intubation. Ischemia was induced by ligating the left anterior descending coronary artery (LAD) using an 8-0 nylon suture with a section of PE-10 tubing placed over the LAD at a position 1 mm from the tip of the normally positioned left atrium. After occlusion for 30 min, reperfusion was initiated by releasing the ligature and removing the PE-10 tubing. After reperfusion for 3 h, the heart was removed and immediately placed in ice-cold PBS. Hearts exhibiting infarcts involving the anterior and apical regions were rapidly frozen and stored in liquid nitrogen (N_2_) for Western blot analysis. In some cases, an entire cross-section was fixed with 4% paraformaldehyde solution for haematoxylin and eosin staining and immunohistochemistry.

### Electron Microscopy

Cells and hearts from all the groups were imaged by transmission electron microscopy (TEM) to observe mitochondrial ultrastructure and mitophagy. The samples were immediately fixed with 2% glutaraldehyde and 2% paraformaldehyde in 0.1 M PBS at 4 °C overnight. After washing with PBS, the samples were postfixed with 2% osmium tetroxide. The fixed samples were dehydrated in a graded alcohol series and embedded in epoxy resin. Ultrathin sections were double-stained with uranyl acetate and lead citrate and then examined with a Hitachi H700 electron microscope (Hitachi, Tokyo, JP).

### Statistical Analysis

The data are presented as the mean ± standard error of the mean (SEM) from at least three independent experiments. One-way analysis of variance (ANOVA) was performed between groups, followed by Dunnett’s post hoc test. *p* < 0.05 indicated statistical significance.

## Results

### Vit D Reduces Apoptotic Cell Death After Hypoxia/Reoxygenation Injury *in vitro*

**FIGURE 1:**
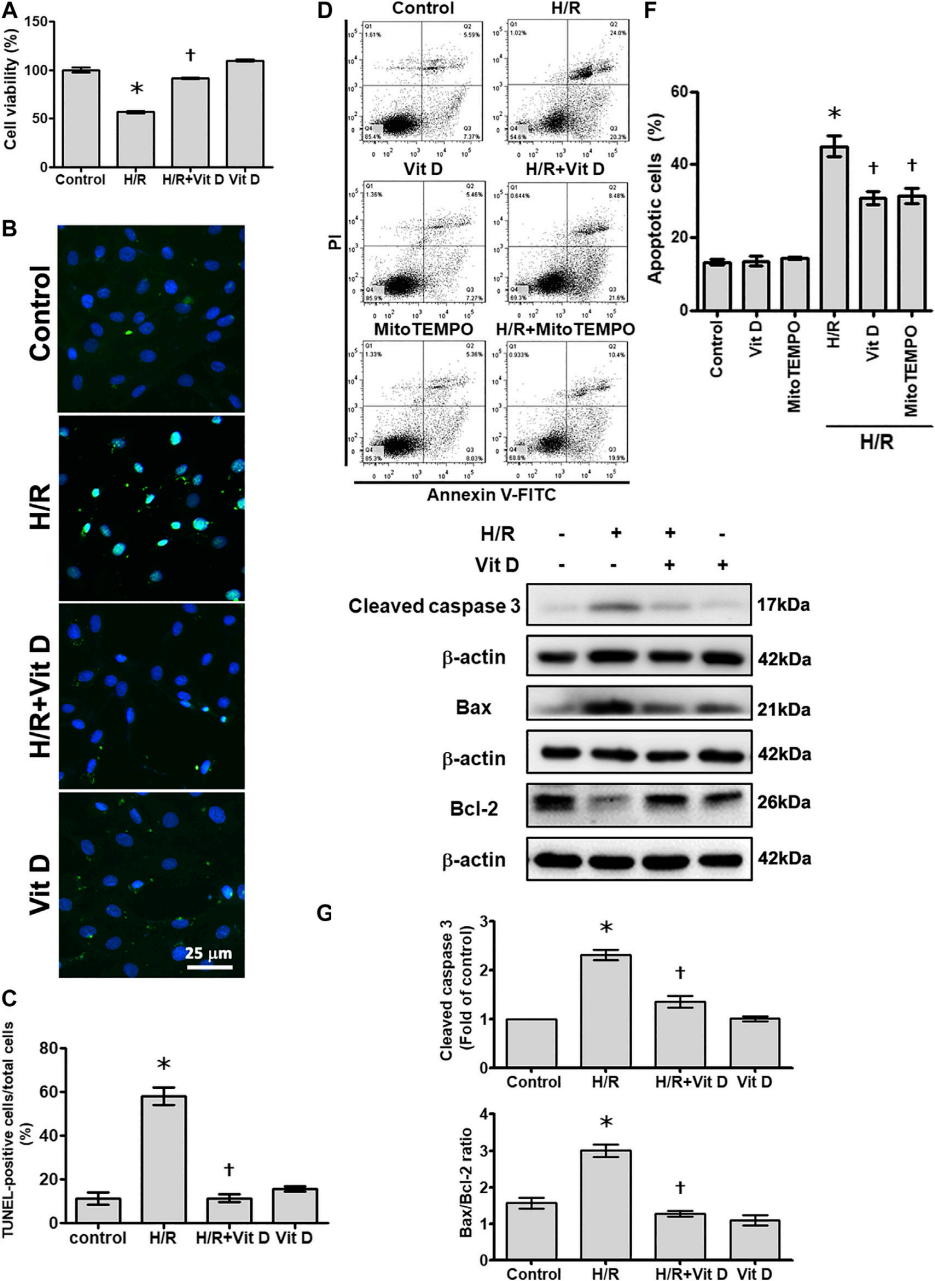
Vitamin D_3_ (25-OH) reduced apoptotic cell death following H/R injury. H9c2 cells were incubated with or without 100 nM Vit D for 4 h and then exposed to hypoxic conditions for 6 h. The cells were then exposed to normoxic conditions for another 12 h. **(A)** Cell viability was measured by MTT assay. (n = 3) **(B and C)** Assessment and calculation of apoptosis by TUNEL assay. The merged images show that apoptotic cells and nuclei appear green and blue, respectively. Scale bar = 25 μm. (n = 4) **(D and E)** Representative images and flow cytometric analysis of the percentage of apoptotic H9c2 cells in the H/R model with or without pretreatment with Vit D or MitoTEMPO by annexin V/PI staining. (n = 4) **(F and G)** The levels of cleaved caspase 3, Bax, and Bcl-2 expression were analyzed by Western blot. The ratio of Bax to Bcl-2 reflects the apoptotic activity (n = 3). *p < 0.05 vs. control, ^†^ *p* < 0.05 vs. H/R.

To evaluate whether Vit D pretreatment improves myocardial cell damage under I/R conditions, H9c2 cells were pretreated with Vit D or PBS and then subjected to H/R *in vitro* to simulate the physiological stress experienced by cardiomyocytes during ischemic infarct. Various concentrations of Vit D were used to treat H9c2 cells for 24 h, and the cell viability, which was evaluated by MTT assay, was not reduced (data not shown). Then, H9c2 cells were incubated with or without 100 nM Vit D for 4 h, exposed to hypoxic conditions for 6 h and treated with reoxygenation for 12 h under normoxic conditions (H/R). The MTT assay showed that H/R treatment significantly decreased H9c2 cell viability, while Vit D pretreatment reduced this effect (Figure 1A). Apoptotic cells were detected using the TUNEL assay and annexin V-FITC/PI staining assay. As shown by TUNEL analysis, H/R treatment significantly increased apoptosis, whereas Vit D pretreatment significantly decreased apoptosis (Figures 1B,C). H/R increased the annexin V-positive apoptotic cell populations (annexin V positive/PI positive and annexin V positive/PI negative), whereas Vit D pretreatment significantly decreased these cell populations (Figures 1D,E). To further confirm that the effects of Vit D on apoptosis are due to its antioxidant activity, MitoTEMPO, a mitochondrial ROS scavenger, was used, and its effect on H/R-induced apoptosis was evaluated. Similar to Vit D, the flow cytometry results indicated that MitoTEMPO also reduced apoptosis in H/R-treated cardiomyocytes (Figures 1D,E). Cleaved caspase three is considered an indicator of apoptosis. Compared to the control, H/R treatment significantly increased the cleaved caspase three levels, while Vit D decreased the levels of cleaved caspase 3 (Figures 1F,G). The expression balance of the BCL-2 families, including pro-apoptotic proteins (such as Bax) and anti-apoptotic proteins (such as Bcl-2), determines cell fate (Siddiqui et al., 2015). We detected the levels of Bcl-2 and Bax expression using Western blot (Figures 1F,G). H/R treatment significantly increased the level of the Bax/Bcl-2 ratio, while Vit D pretreatment decreased it.

### Vit D Inhibits Reactive Oxygen Species Production in Hypoxia/Reoxygenation-Treated Cardiomyocytes

**FIGURE 2:**
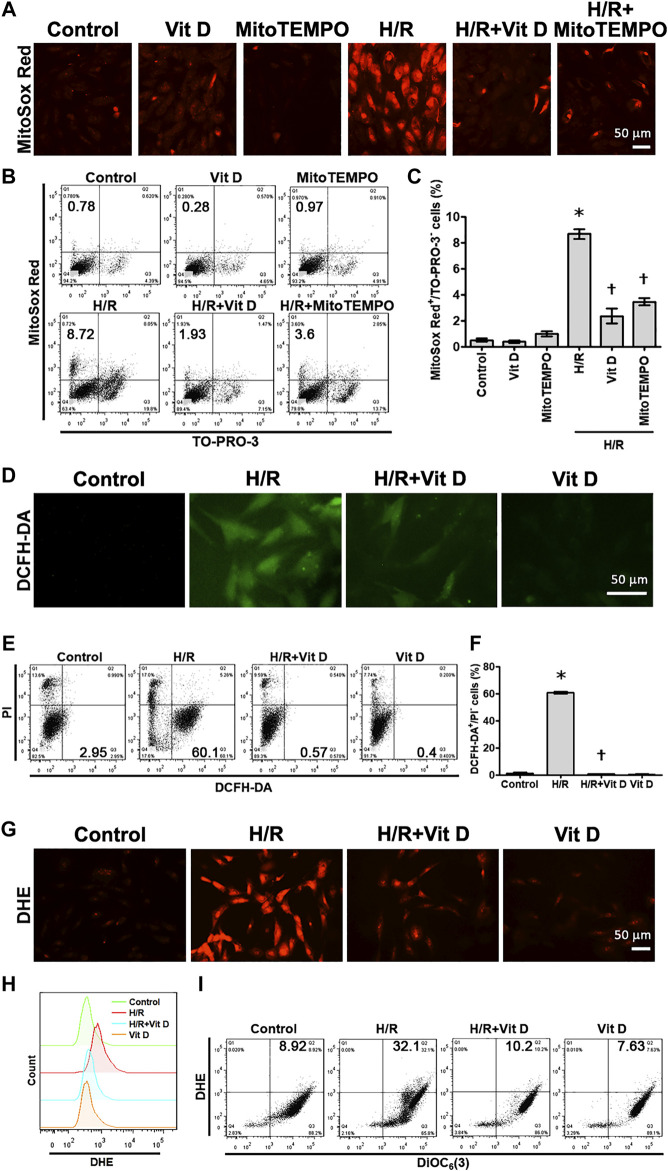
Vit D inhibited ROS production in H/R-treated cardiomyocytes. Cardiomyocytes were incubated with or without 100 nM Vit D for 4 h and then exposed to hypoxia for 6 h. The cells were then exposed to normoxia for another 12 h **(A and B, C)** MitoSox Red was used to detect mitochondrial ROS by fluorescence microscopy. MitoSox Red/TO-PRO-3 staining was used to distinguish viable cells producing mitochondrial ROS (MitoSox Red-positive/TO-PRO-3-negative) by flow cytometry. **(D and E, F)** DCFH-DA was used to detect cytoplasmic H_2_O_2_ by fluorescence microscopy. DCFH-DA/PI staining was used to examine viable cells with cytoplasmic H_2_O_2_ (DCFH-DA-positive/PI-negative) by flow cytometry. **(G)** DHE was used to detect cellular superoxide anions by fluorescence microscopy. **(H)** DHE fluorescence displayed by overlay signals using flow cytometry. **(I)** DHE/DiOC_6_(3) staining was used to detect viable cells with cellular superoxide anions (DHE-positive/DiOC_6_(3)-positive) by flow cytometry. Scale bar = 50 μm. (n = 3, *p < 0.05 vs. control, ^†^ *p* < 0.05 vs. H/R).

To investigate whether Vit D can affect mitochondrial ROS production in cardiomyocytes under H/R conditions, MitoSox Red was used to detect mitochondrial ROS. The results showed that H/R treatment significantly increased the production of mitochondrial ROS, while Vit D pretreatment decreased this effect (Figure 2A). Similar to pretreatment with Vit D, treatment with MitoTEMPO, a mitochondria-specific ROS scavenger, significantly decreased the mitochondrial ROS production in viable cells (MitoSox Red-positive and TO-PRO-3-negative) after H/R injury (Figures 2B,C). In addition, the cytoplasmic superoxide anion and H_2_O_2_ levels were detected using fluorescent protein-based redox probes, namely, DCFH-DA and DHE, respectively. H/R treatment significantly increased the cellular production of cytoplasmic superoxide anions and H_2_O_2_, as determined by fluorescence microscopy, while Vit D pretreatment reversed these effects (Figures 2D,G). In addition, H/R treatment increased the production of cytoplasmic superoxide anions in viable cells (PI-negative and DCFH-DA-positive), as shown by flow cytometry, while Vit D significantly decreased it (Figures 2E,F). Interestingly, the Vit D-treated group had lower DCFH-DA levels than the control group, and the H/R + Vit D group also had lower DCFH-DA levels than the control group. Additionally, the DHE overlay showed that H/R increased cytoplasmic H_2_O_2_, while Vit D decreased cytoplasmic H_2_O_2_ (Figure 2H). H/R increased the cytoplasmic H_2_O_2_ in viable cells (DHE-positive and DiOC_6_(3)-positive), as shown by flow cytometry, whereas Vit D decreased it (Figure 2I). These results indicate that Vit D is an effective antioxidant in cardiomyocytes.

### Vit D Attenuates the Hypoxia/Reoxygenation-Induced Reduction in the Mitochondrial Membrane Potential (ΔΨm) and ATP Levels

**FIGURE 3:**
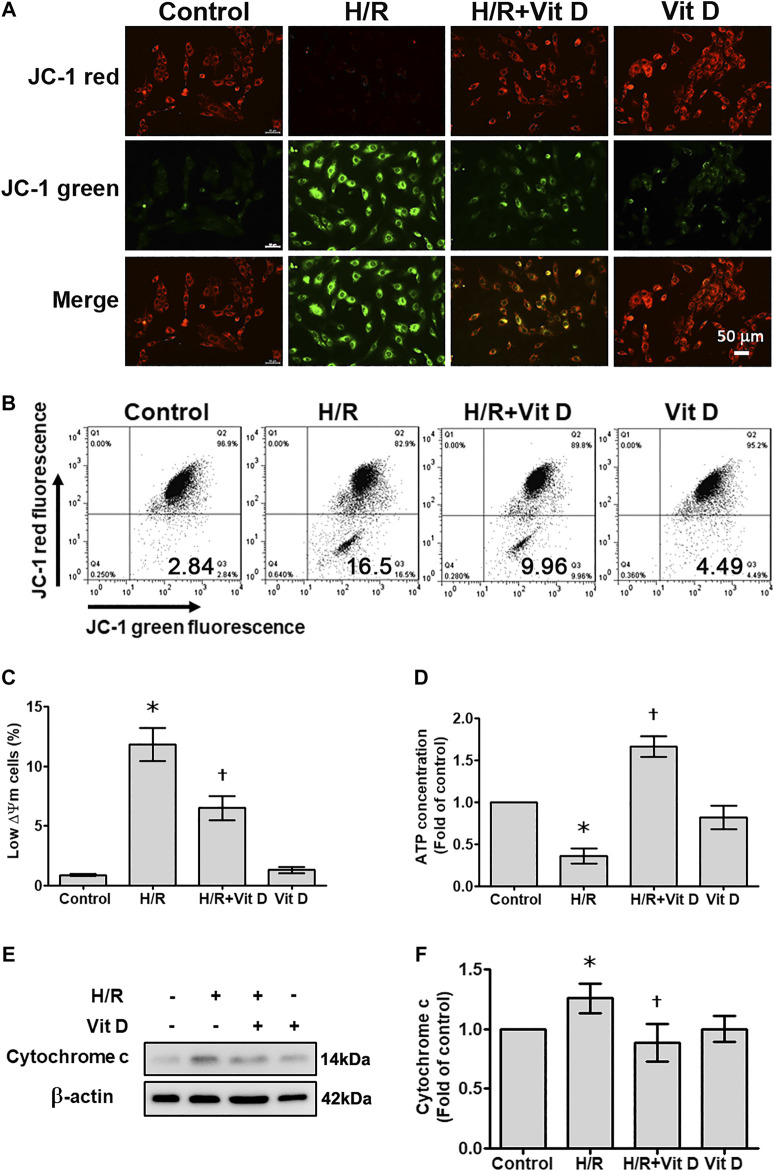
Vit D restored the mitochondrial membrane potential and ATP production that were decreased by H/R. Cardiomyocytes were incubated with or without 100 nM Vit D for 4 h and then exposed to hypoxia for 6 h. The cells were then exposed to normoxia for another 12 h. **(A)** Representative fluorescence images of JC-1 staining show the change in high mitochondrial membrane potential (red) and low mitochondrial membrane potential (green), which is an early event of apoptosis. H/R treatment increased green fluorescence, whereas Vit D decreased green fluorescence. Scale bar = 50 μm. **(B and C)** Flow cytometric patterns and quantitative analysis of cardiomyocytes stained with JC-1. Cell population with low ΔΨm according to JC-1 staining (green positive and red negative) (n = 4). **(D)** ATP levels were decreased after H/R injury, but ATP levels were restored by Vit D treatment. **(E)** The expression of cytochrome c was detected by Western blot. (n = 3). *p < 0.05 vs. control, ^†^ *p* < 0.05 vs. H/R.

The ΔΨm and ATP levels serve as critical parameters for cell apoptosis and mitochondrial function. In most cardiomyocytes, H/R induced marked changes in terms of a shift in fluorescence emission from red to green, as shown by the JC-1 assay; this shift indicated ΔΨm dissipation. The emission of green fluorescence by cardiomyocytes pretreated with Vit D was reduced, and the distribution pattern was similar to that of the control group. In addition, the results of JC-1 analysis by flow cytometry revealed that H/R treatment increased the populations of cells with low ΔΨm (JC-1 green positive and red negative), whereas Vit D reversed this effect (Figures 3A–C). The ATP content was decreased after H/R injury, while Vit D treatment protected cardiomyocytes from this H/R-induced effect (Figure 3D). The ATP concentration of the H/R + Vit D group was higher than that of the control group. The dissipation of ΔΨm can lead to mitochondria-dependent apoptosis. In addition, H/R induced the expression of cytochrome c, a marker of mitochondrial apoptosis, whereas this upregulation of cytochrome c was reduced by Vit D pretreatment (Figures 3E,F). These results indicate that the protective effect of exogenous Vit D on cardiomyocytes occurs via the modulation of mitochondrial function.

### Vit D Protects Mitochondria by Reducing Mitochondrial Fission

**FIGURE 4:**
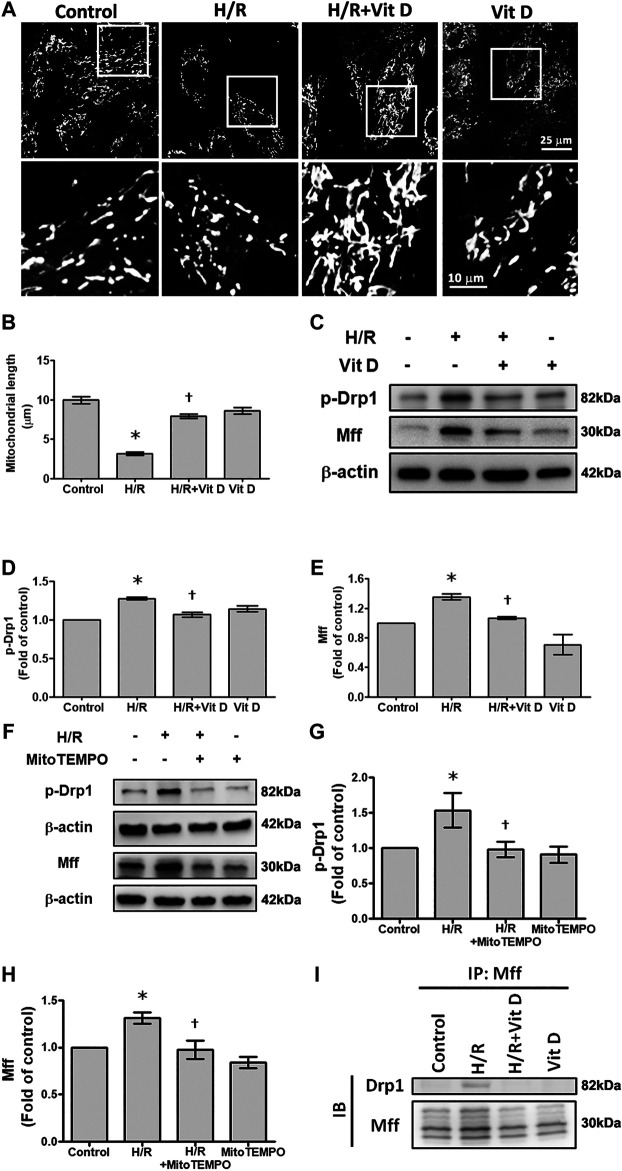
Vit D reduced mitochondrial fission in H/R-treated cardiomyocytes. **(A and B)** The mitochondrial morphology in cardiomyocytes was measured by the MitoTracker assay. Scale bar = 25 or 10 μm, as indicated in the panel. **(C–E)** Mitochondrial fission-associated regulatory factors p-Drp1 and Mff were detected by Western blot (n = 5). **(F–H)** Measurement of the effect of MitoTEMPO treatment on the expression of p-Drp1 and Mff by Western blot (n = 3). **(I)** Measurement of the interaction of Drp1 and Mff by co-immunoprecipitation. The indicated cultured cells were immunoprecipitated with anti-Drp1 antibodies followed by immunoblotting with anti-Mff antibodies. *p < 0.05 vs. control, ^†^ *p* < 0.05 vs. H/R.

Mitochondria are highly dynamic organelles that undergo fission and fusion, and these processes are closely related to mitochondria function and ROS production (Westermann, 2010). MitoTracker was used to examine the effects of Vit D on the mitochondrial morphology in H/R-treated cardiomyocytes. The results showed that the length of the mitochondria in H/R-treated cells was significantly shorter than that in control-treated cells, and Vit D reversed this effect (Figures 4A,B). To further investigate how Vit D regulates mitochondrial fission, p-Drp1 and Mff, two regulatory proteins associated with mitochondrial fission, were examined. Our results showed that H/R treatment increased the expression of p-Drp1 and Mff, whereas the expression of these proteins was decreased by Vit D (Figures 4C–E). To further confirm the effects of mitochondrial ROS on mitochondrial fission after H/R injury, we examined the expression of p-Drp1 and Mff in Mito TEMPO and H/R-treated cardiomyocytes. We found that MitoTEMPO reduced the expression of both p-Drp1 and Mff (Figures 4F–H). Next, we investigated whether Drp1 interacts with Mff. We found that Drp1 was co-immunoprecipitated with Mff following H/R treatment, while Vit D decreased the interaction (Figure 4I). Our results suggest that the protective effect of Vit D against H/R-induced mitochondrial fission is attributed to its antioxidant capacity.

### Vit D Reduces Mitophagy in Cardiomyocytes

**FIGURE 5:**
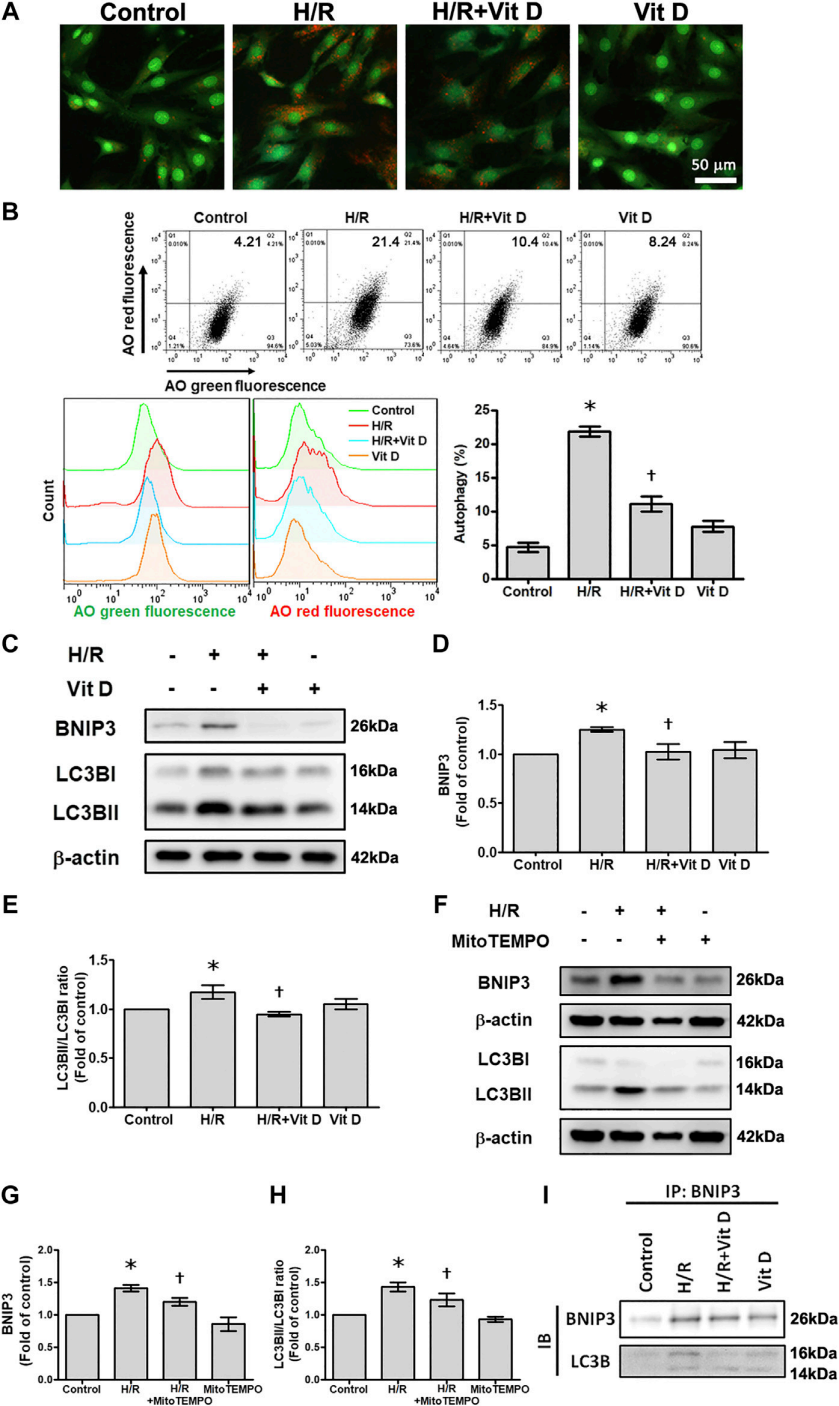
Vit D attenuated H/R-induced mitophagy in cardiomyocytes. **(A)** Cells were stained with acridine orange (AO) solution and observed under a fluorescence microscope to examine the changes in the acidic compartments of the cells. Scale bar = 50 μm. **(B)** Cells were stained with AO solution and analyzed by flow cytometry. AO green and red overlay fluorescence showed that H/R enhanced AO staining, whereas Vit D reduced it. The bar graph shows the percentages of cells undergoing autophagy (AO red positive and green positive) (n = 3). **(C–E)** Expression of BNIP3 and LC3BII/I was examined by Western blot (n = 5). **(F–H)** Measurement of the effects of MitoTEMPO on the expression of BNIP3 and LC3BII/I by Western blot (n = 5). **(I)** Measurement of the interaction of BNIP3 and LC3BII/I by co-immunoprecipitation. The indicated cultured cells were immunoprecipitated with anti-BNIP3 antibodies followed by immunoblotting with anti-LC3B antibodies. *p < 0.05 vs. control, ^†^ *p* < 0.05 vs. H/R.

It has been reported that mitophagy plays a regulatory role in apoptosis (Yussman et al., 2002). Acridine orange (AO) staining was used to detect autophagic vacuoles. H/R-treated cells exhibited increased autophagosome formation, which was inhibited when cells were pretreated with Vit D; these results indicated that Vit D reduced the accumulation of autophagosomes (Figure 5A). In addition, the results of AO analysis by flow cytometry suggested that H/R injury significantly increased the population of cells undergoing autophagy (AO green positive and red positive), while Vit D decreased this population. AO green and red fluorescence overlay showed that H/R treatment enhanced AO staining, whereas Vit D reduced AO staining (Figure 5B). To investigate how Vit D affects mitophagy, we analyzed the expression of BNIP3 and the conversion of LC3I to LC3II by Western blot. The results showed that H/R increased BNIP3 expression and the LC3II/LC3I ratio. Nevertheless, Vit D reduced the expression of both BNIP3 and LC3BII (Figures 5C–E). To further confirm the effects of mitochondrial ROS on mitophagy after H/R injury, we examined the expression of BNIP3 and the conversion of LC3I to LC3II in MitoTEMPO and H/R-treated cardiomyocytes. We found that MitoTEMPO reduced the expression of both BNIP3 and LC3BII/LC3BI ratio (Figures 5F–H). Next, we investigated whether BNIP3 interacts with LC3B. Strong interactions between BNIP3 and LC3B were observed in H/R-treated cardiomyocytes, and the addition of Vit D markedly inhibited this interaction (Figure 5I). Taken together, these results indicate that Vit D can attenuate H/R-induced mitophagy by reducing mitochondrial ROS production.

### Vit D Reduces Mitochondrial Translocation of Drp1, Mff, BNIP3, and LC3B After Hypoxia/Reoxygenation Treatment

**FIGURE 6:**
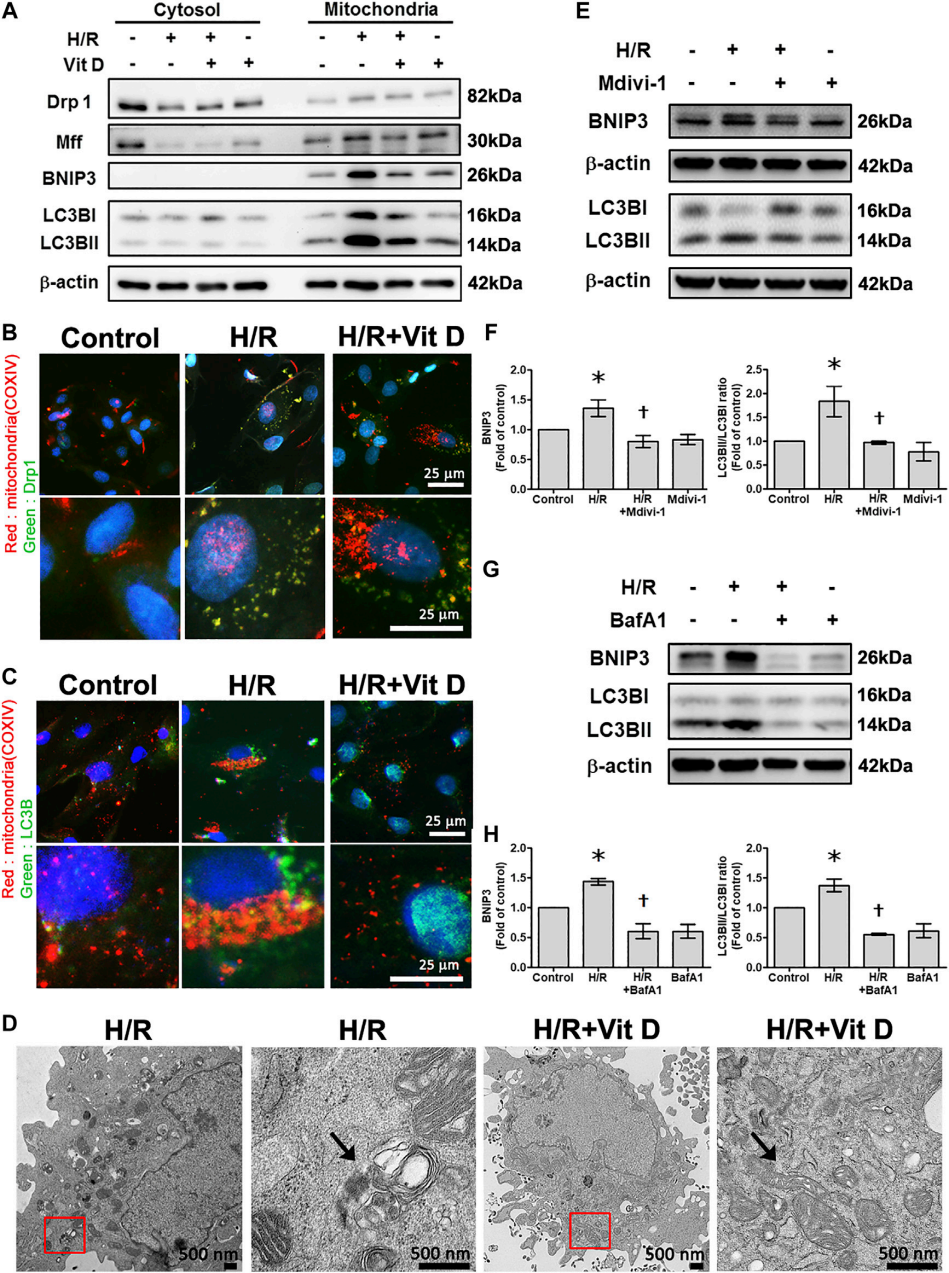
Vit D reduced the translocation of mitochondrial fission-associated proteins (Drp1 and Mff) and mitophagy-associated proteins (BNIP3 and LC3B) in H/R-treated cardiomyocytes. **(A)** Mitochondrial translocation of Drp1, Mff, BNIP3, and LC3B in response to H/R treatment. H/R treatment induced a significant increase in Drp1, Mff, BNIP3 and LC3B translocation to mitochondria. Vit D treatment reduced this translocation. **(B and C)** Co-localization of COX IV (a mitochondrial marker) and Drp1 or LC3B was examined by dual immunofluorescence staining. The co-localization was increased in the H/R group compared to the control group, while Vit D treatment reduced this effect. Scale bar = 25 μm. **(D)** TEM showed that autophagic vacuoles (arrow) were present in the H/R-treated cells, and Vit D reduced the presence of autophagic vacuoles. The right panels are the magnification of the region enclosed in the red box. Scale bar = 500 nm. **(E and F)** The effect of Mdivi-1 (an inhibitor of mitochondrial fission) on the expression levels of BNIP3 and LC3BII/LC3BI ratio were analyzed by Western blot. LC3BII/LC3BI ratios reflect the autophagy activity (n = 3). **(G and H)** The effects of bafilomycin A1, an autophagy inhibitor, on the expression levels of BNIP3 and LC3BII/I ratio were analyzed by Western blot (n = 3). *p < 0.05 vs. control, ^†^ *p* < 0.05 vs. H/R.

We then further evaluated the mitochondrial translocation of Drp1, Mff, BNIP3, and LC3B in response to H/R treatment. H/R treatment induced a significant increase in Drp1, Mff, BNIP3 and LC3B translocation to the mitochondria. However, Vit D treatment reduced the translocation of these proteins (Figure 6A). Using double immunofluorescence staining, we further investigated changes in the mitochondria undergoing mitophagy. We observed that co-localization of COX IV (a mitochondrial marker) with Drp1 or LC3B was more clearly present in the H/R group than in the control group, while Vit D pretreatment reduced these phenomena (Figures 6B,C). Transmission electron microscopy images showed that Vit D reduced the presence of autophagic vacuoles in H/R-treated cardiomyocytes (Figure 6D). To further evaluate the effect of the mitochondrial fission on H/R-induce mitophagy, we performed the treatment of mitochondrial fission inhibitor (Mdivi-1) on the expression of mitophagy-related proteins in H/R-treated H9c2 cells. Cells with Mdivi-1 treatment significantly reduced the levels of H/R-induced BNIP3 and LC3BII/LC3BI ratio expression (Figure 6E,F). These results indicated that mitochondrial fission was involved in H/R-induced mitophagy. Furthermore, we examined the effects of Vit D on HR-induced cardiomyocyte injury via mitophagy, we performed the effect of bafilomyocin A1, an autophagy inhibitor, on the expression of mitophagy-related proteins. Cells with bafilomyocin A1 treatment significantly reduced H/R-induced BNIP3 and LC3BII/LC3BI expression (Figures 6G,H).

### Vit D Preserves Mitochondrial Membrane Integrity and Ultrastructure in Mouse Hearts After Ischemia/Reperfusion Injury

**FIGURE 7:**
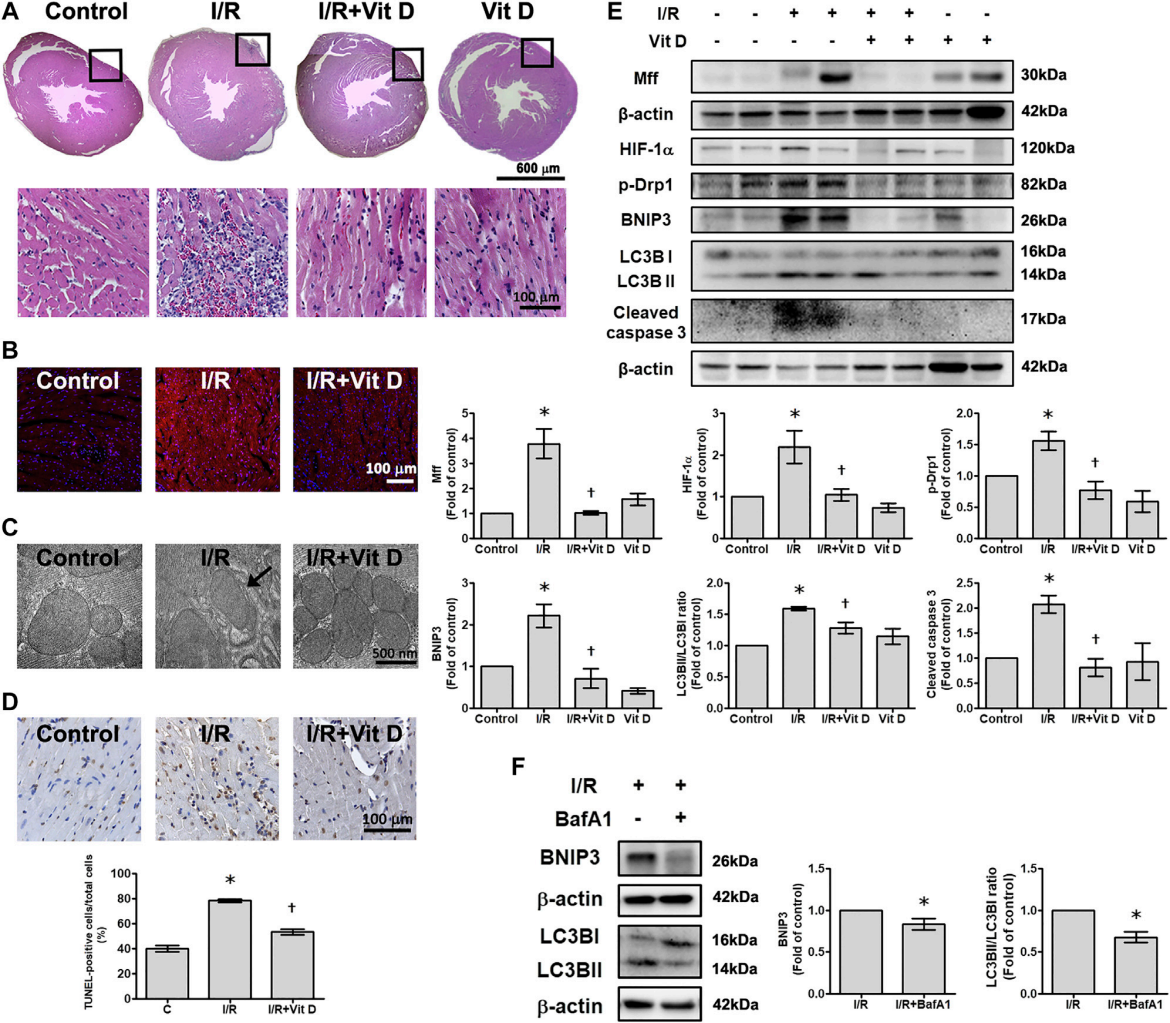
Vit D reduced I/R-induced cardiac injury. **(A)** Transverse sections of cardiac morphology were observed by haematoxylin and eosin staining. I/R induced more inflammatory cell infiltration into cardiac tissue than the control, while Vit D ameliorated this inflammatory infiltration. Scale bar = 600 μm or 100 μm, as indicated in the panel. **(B)** The production of superoxide anions was assessed by DHE staining. Scale bar = 100 μm. **(C)** Representative ultrastructural images were observed by TEM. Mitophagy was present in the I/R-treated cardiomyocytes. The control and Vit D treatment groups exhibited intact mitochondrial structures with well-arranged cristae. Scale bar = 500 nm. **(D)** Myocardial apoptosis was identified and quantified by TUNEL assay (TUNEL: brown; nuclei: blue; scale bar = 100 μm). **(E)** The protein levels of HIF-1α, Drp1, Mff, BNIP3, LC3B, and cleaved caspase three were determined by Western blot. Vit D treatment significantly reduced the levels of these proteins in the I/R-treated mice. **(F)** The effect of bafilomyocin A1, an autophagy inhibitor, on the levels of BNIP3 and LC3BII/I expression in I/R-treated mice (n = 3). *p < 0.05 vs. control, ^†^ *p* < 0.05 vs. I/R.

Compared with sham surgery, I/R induced the infiltration of inflammatory cells into the infarct zone of cardiac tissues, and Vit D ameliorated this inflammatory infiltration (Figure 7A). I/R increased the intracellular levels of superoxide anions, as shown by DHE staining, while Vit D decreased the intercellular levels of superoxide anions in the heart after I/R injury (Figure 7B). Ultrastructure images were examined by TEM. The mice administered Vit D treatment showed complete mitochondrial structures with well-aligned cristae (Figure 7C). I/R treatment significantly increased the number of apoptotic cells, while Vit D pretreatment reduced the effect (Figure 7D). The levels of HIF-1α, Mff, p-Drp 1, BNIP3, LC3B and cleaved caspase three in the I/R-treated mice were significantly elevated compared to those in the control-treated mice. Furthermore, Vit D treatment significantly reduced the levels of these proteins during I/R damage (Figure 7E). Furthermore, we examine the effects of Vit D on I/R-induced mitophagy, we performed the effect of bafilomyocin A1 on the expression of mitophagy-related proteins. Mice with bafilomyocin A1 treatment significantly reduced I/R-induced BNIP3 and LC3BII/I expression (Figure 7F).

## Discussion

The important findings we report are that exogenous Vit D treatment can 1) reduce cardiomyocyte apoptosis and ROS production; 2) increase mitochondrial membrane potential; 3) reduce mitochondrial fission via decreased Drp1 and Mff expression; and 4) reduce mitophagy by reducing the H/R-induced expression of BNIP3 and LC3B. Vit D exerts a protective effect on mitochondrial physiology and morphology and on the expression of key mitochondrial proteins. To the best of our knowledge, this study is one of few studies to show that Drp1/Mff is required for mitochondrial fission and that BNIP3/LC3B is associated with mitophagy; these findings further elucidate the mechanisms of cardiac I/R injury and the effective protection provided by Vit D.

Vit D exerts a wide range of physiological and cytoprotective functions in biological systems. Human skeletal muscle cells treated with 1α,25-dihydroxyvitamin D_3_ exhibited an increased mitochondrial oxygen consumption rate and mitochondrial volume (Ryan et al., 2016). 25(OH)D_3_ and 1α,25(OH)_2_D_3_ affected calcium and phosphorus absorption, gene expression, proliferation, and differentiation in skeletal muscle cells (Birge and Haddad, 1975; Girgis et al., 2014). Vit D depletion exacerbated hypertension and cardiac damage (Andersen et al., 2015). As reported in the literature, Vit D can alleviate H_2_O_2_-mediated endothelial cell stress-mediated injury in a dose- and time-dependent manner by reducing anionic superoxide production and apoptosis (Polidoro et al., 2013). Vit D prevents the inflammatory responses observed in endothelial cells and db/db mice exposed to diabetic conditions (Einbinder et al., 2016). Vit D helps reduce diabetic cardiomyopathy not only by improving blood sucrose and insulin levels but also by downregulating advanced glycation end product formation and hexosamine pathways in heart tissues (Derakhshanian et al., 2019). Activation of the Vit D receptor prevents myocardial reperfusion injury by inhibiting apoptosis and modulating autophagy (Yao et al., 2015). In accordance with these findings, in the present study, Vit D significantly attenuated I/R-induced cardiac apoptosis *in vivo* and *in vitro*.

During the pathogenesis of myocardial I/R injury, oxidative stress is increased and contributes to the regulation of cardiomyocyte apoptosis (Chen and Zweier, 2014; Zhou et al., 2015). Vit D, an antioxidant vitamin, is essential in regulating the biochemical pathways that lead to proper heart function (Liu et al., 2018). Consistent with these findings, we demonstrated that H/R treatment increased ROS production. Furthermore, Vit D reduced cytoplasmic and mitochondrial ROS production. Mitochondria are the main source of ROS, and excessive ROS production severely accelerates mitochondrial abnormalities and mitochondrial membrane injury (Bliksoen et al., 2015; Ong et al., 2015). Importantly, we demonstrated that the specific mitochondrial ROS scavenger MitoTEMPO has the same effect on ROS production as Vit D, indicating that in H/R-treated cardiomyocytes, cytoplasmic ROS are mainly derived from mitochondrial ROS. Therefore, we concluded that the antioxidant effects of Vit D may be attributed to the removal of excess mitochondrial ROS. Cardiomyocytes treated with H/R exhibited mitochondrial dysfunction, as evidenced not only by the decrease in both ATP production and mitochondrial membrane potential but also by the increase in ROS production in the present study. These H/R-induced functional and structural defects in mitochondria are associated with mitochondria-dependent apoptotic events, including caspase three activation and chromosomal DNA fragmentation. Importantly, we demonstrated that Vit D prevents H/R-induced cardiac apoptosis by restoring mitochondrial function and regulating intracellular redox status.

Mitochondria are essential for cardiomyocyte survival and death (Yang et al., 2018). Healthy mitochondria produce ATP energy that drives biological processes, while damaged mitochondria produce pathological ROS (Tang et al., 2015). To prevent cell death and maintain mitochondrial function, damaged mitochondria may undergo fission and degradation. Oxidative stress regulates mitochondrial fission events (Kroemer et al., 2007). Mitochondrial fission usually results in the separation of components from an original mitochondrion, producing one healthy mitochondrion and another impaired mitochondrion with reduced membrane potential (Youle and van der Bliek, 2012). However, oxidative stress may lead to excessive mitochondrial fission, resulting in mitochondrial structural changes and dysfunction as well as cellular damage (Reddy, 2014). MitoTracker and transmission electron microscopy revealed mitochondrial rupture or smaller mitochondria in H/R-treated cardiomyocytes, indicating that mitochondrial fission was increased and that Vit D improved this effect. The effect of MitoTEMPO on mitochondrial morphology was identical to that of Vit D, suggesting that the effect of Vit D on mitochondrial fission could be attributed to its antioxidant activity. In addition, mitochondrial fission is regulated by proteins including Drp1 and Mff (Westermann, 2010; Jin et al., 2018; Jin et al., 2020). Drp1 is a cytosolic guanosine triphosphatase that plays an important role in translocation and oligomerization in mitochondrial outer membranes during mitochondrial fission (Hall et al., 2014). Overexpression of a dominant-negative form of Drp1 inhibits cytochrome c release and caspase three activation in H9c2 cells treated with high glucose (Yu et al., 2008). The levels of the Dynamin two and Drp1 proteins were significantly increased when cardiomyocytes were exposed to 30 μM H_2_O_2_ (Gao et al., 2016). It is known that the inhibition of Drp1 can reduce the death of cardiomyocytes (Ong et al., 2010). I/R injury increased ROS production and mitochondrial fission in cardiac microvascular endothelial cells (Zhou et al., 2019; Wang et al., 2020). Our study showed that H/R treatment could upregulate the expression of p-Drp1 and Mff, while Vit D could reverse this effect. Furthermore, in this study, when mice were exposed to I/R, apoptosis, caspase three activity, and mitochondrial fission-associated protein levels exhibited the same trends as those observed *in vitro*. Our data suggested that Vit D can inhibit mitochondrial fission, thereby protecting the heart from I/R injury.

Autophagy is a lysosome-dependent cellular catabolic process that is accompanied by LC3B transformation and BNIP3 expression; autophagy results in the timely clearance of damaged cellular components (Yang and Klionsky, 2010). Maintaining a balance between mitophagy, a selective form of autophagy, and mitochondrial production is critical for maintaining healthy cells (Livingston et al., 2019). Experiments over the years have shown different results, and the effects of mitophagy on diseases associated with organ ischemia is a controversial topic (Andres et al., 2015; Li et al., 2016; Zhou et al., 2018a; Zhou et al., 2018b; Zhang et al., 2018; Wu et al., 2019). Different cell types, animal species, and I/R models can yield to contradictory results. In our study, H/R treatment significantly increased mitophagy by increasing the LC3 -II B/LC3B -I levels and BNIP3 expression compared with the sham operation. In addition, using acridine orange staining and transmission electron microscopy, we observed that H/R significantly induced autophagosome formation. Furthermore, we showed by immunocytochemistry and confocal microscopy analysis that COX IV-labelled mitochondria colocalized with LC3B-labelled autophagosomes, indicating the presence of mitophagy in H/R-treated cardiomyocytes. These features of mitophagy are significantly activated by H/R injury in cardiomyocytes and can be further attenuated by Vit D treatment or MitoTEMPO treatment or Mdivi-1 treatment. H/R damage to cardiomyocytes significantly activates these characteristics of mitochondria, which can be reduced by Vit D treatment or MitoTEMPO treatment or Mdivi-1 treatment. This study describes a new mechanism by which Vit D exerts cardioprotective effects by reducing mitophagy-mediated cell death under I/R conditions. The inhibitory effect of Vit D on ROS production may be due in part to decreased mitophagy. Furthermore, when mice were exposed to I/R, the levels of mitophagy-associated proteins showed the same trends as those observed *in vitro*. In summary, these results suggested that a decrease in mitophagy is associated with the cardioprotective effects of Vit D during cardiac I/R injury.

Taken together, our current study shows that Vit D prevents H/R-induced apoptosis by inhibiting oxidative stress and regulating mitochondrial function. Vit D inhibits H/R-induced mitochondrial fission and mitophagy by inhibiting Drp1/Mff and BNIP3/LC3B, respectively. After describing the mechanisms of Vit D-mediated protection, this study provides new opportunities for treating H/R-induced cardiac damage.

## Data Availability Statement

The original contributions presented in the study are included in the article/Supplementary Material, further inquiries can be directed to the corresponding authors.

## Ethics Statement

The animal study was reviewed and approved by National Institutes of Health guidelines for the use of laboratory animals and approved by the Taiwan University Animal Ethics Committee.

## Author Contributions

TLL and MHL contributed equally as first authors. TLL, YCC, YCL, and TCL performed the experiments. MHL, YHL, LFH, and HCS contributed to the data analysis. CWL and YLC conceived and coordinated the study and wrote the manuscript. All the authors approved the final version to be published.

## Funding

This research was supported in part by research grants from the Ministry of Science and Technology of Taiwan (MOST 108-2320-B002-065-MY3), Chang Gung Medical Research Program Foundation (CMRPG6J0221, 22, 23), and Kaohsiung Medical University Hospital (kmtth-105-054).

## Conflict of Interest

The authors declare that the research was conducted in the absence of any commercial or financial relationships that could be construed as a potential conflict of interest.
